# Unlocking higher harmonics in atomic force microscopy with gentle interactions

**DOI:** 10.3762/bjnano.5.29

**Published:** 2014-03-11

**Authors:** Sergio Santos, Victor Barcons, Josep Font, Albert Verdaguer

**Affiliations:** 1 Departament de Disseny i Programació de Sistemes Electrònics, UPC - Universitat Politècnica de Catalunya Av. Bases, 61, 08242 Manresa (Barcelona), Spain; 2 ICN2 - Institut Catala de Nanociencia i Nanotecnologia, Campus UAB, 08193 Bellaterra (Barcelona), Spain; 3 CSIC - Consejo Superior de Investigaciones Cientificas, ICN2 Building ,08193 Bellaterra (Barcelona), Spain

**Keywords:** atomic force microscopy, chemistry, composition, heterogeneity, higher harmonics, phase

## Abstract

In dynamic atomic force microscopy, nanoscale properties are encoded in the higher harmonics. Nevertheless, when gentle interactions and minimal invasiveness are required, these harmonics are typically undetectable. Here, we propose to externally drive an arbitrary number of exact higher harmonics above the noise level. In this way, multiple contrast channels that are sensitive to compositional variations are made accessible. Numerical integration of the equation of motion shows that the external introduction of exact harmonic frequencies does not compromise the fundamental frequency. Thermal fluctuations are also considered within the detection bandwidth of interest and discussed in terms of higher-harmonic phase contrast in the presence and absence of an external excitation of higher harmonics. Higher harmonic phase shifts further provide the means to directly decouple the true topography from that induced by compositional heterogeneity.

## Introduction

It has long been recognized in the community that higher harmonics encode detailed information about the non-linearities of the tip–sample interaction in dynamic atomic force microscopy (AFM) [1–5]. Physically, non-linearities relate to the chemical and mechanical composition [6] of the tip–sample system and imply that higher harmonics can be translated into conservative and dissipative [7] nanoscale and atomic properties [8]. Furthermore, conventional dynamic AFM can already reach molecular [9–10], sub-molecular [11] and atomic [12–13] resolution in some systems. Thus, the simultaneous detection and interpretation of multiple higher harmonic signals while scanning [14] can lead to spectroscopy-like capabilities [15–16], such as chemical identification, with similar or higher resolution [5,17–18]. The higher harmonic approach however, and particularly in other than highly damped environments [19–20], requires dealing with the recurrent challenge of detecting higher harmonics [1,3,21–22]. Higher harmonics are a result of the non-linear tip–sample interaction in the sense that the interaction effectively acts as the driving force of each harmonic component [7]. Accordingly, relatively high peak forces, of the order of 1–100 nN, are required [22–23] to excite higher harmonics above the noise level. In order to address this issue, in 2004 Rodriguez and García [23] proposed to drive the second higher flexural mode of the cantilever with an external drive. In this way, and by driving with sufficiently small (sub-nanometer) second mode amplitudes, the first mode amplitude [24] or frequency [17] can be employed to track the sample in amplitude or frequency modulation (AM and FM), respectively. The second mode can then be left as an open loop for high sensitivity mapping of compositional variations [25] or as a closed loop, in which case the tip–sample stiffness *k*_ts_ can be computed [17,26]. More recently, the multifrequency AFM approach has been extended to employ three flexural modes [27] and/or simultaneous torsional modes [28], for which, typically, the frequency and mode under consideration are externally excited [24]. In summary, FM and/or AM feedback systems can be employed in one [29], several [27] or all of the modes under consideration in order to quantify properties on the nanoscale through observables [30] while simultaneously enhancing sensitivity and throughput [31]. The dynamics in the multifrequency approach, however, might lead to extra complexities in the analysis, acquisition and interpretation of data [31–32]. For example, recent studies [31] show that multiple regimes of operation might follow depending on the relative kinetic energy between the higher mode of choice and the fundamental eigenmode [31,33].

Here, exact multiple harmonics of the fundamental drive frequency are externally excited above the noise level to open multiple contrast channels that are sensitive to compositional variations. The focus is on amplitude modulation (AM) AFM, in which the fundamental amplitude A_1_ ≡ A tracks the sample as usual. For standard cantilevers the eigenmodes are nonharmonic [29]. That is, the natural resonant frequencies of the cantilevers are not integer multiples. Furthermore, these natural frequencies relate to the geometry and mechanical properties of the cantilever [34]. The practical implication is that it is only easy to induce large oscillations at the frequencies that coincide with these natural frequencies. Nevertheless the tip–sample coupling always occurs via harmonic frequencies. This is because a periodic motion always implies that there is a fundamental frequency and that all other higher frequencies are integer multiples of the fundamental [35]. The implication is that externally introducing frequencies other than harmonic frequencies could induce a fundamental sub-harmonic frequency [24,35]. In short, the incommensurability between external drives in the standard multifrequency approach implies that the cantilever motion is not exactly periodic relative to the fundamental drive and that a sub-harmonic excitation typically follows [32]. Furthermore, simplifications in eigenmode frequency shift theory [36] might lead to inconsistencies [37]. This issue becomes more prominent when dealing with third or higher eigenmodes [27,38], for which the theory is now emerging [31]. The introduction of exact harmonic external drives keeps the fundamental frequency intact and the analytical expressions are simplified by orthogonality. Furthermore 2(*N*−1) observables, i.e., higher harmonic amplitudes and phases, are made available even with peak forces no higher than 200 pN, as they are required [25,39] for high resolution and minimally invasive imaging of soft matter. Thermal fluctuations are also considered here in order to establish a possible loss of contrast due to fundamental sources of noise. It is also shown that true topography and apparent topography, which is induced by chemical heterogeneity, can be decoupled at once by monitoring the phase contrast of higher harmonics.

## Results and Discussion

Consider the equation of motion of the *m*th eigenmode

[1]
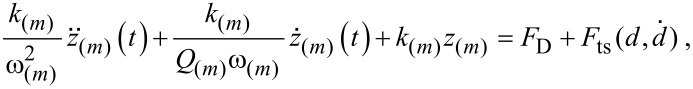


where *k*_(_*_m_*_)_, *Q*_(_*_m_*_)_, ω_(_*_m_*_)_, and z_(_*_m_*_)_ are the spring constant, quality factor, natural frequency and position of the *m*th eigenmode. The term *F*_D_ stands for the external driving force

[2]
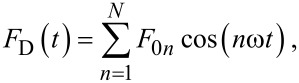


where the subscript without brackets, *n*, indicates the harmonic number. Note that here ω*_n_* = *n*ω, where ω is the fundamental drive frequency set near mode *m* = 1, i.e., ω = ω_1_ ≡ ω_(1)_. The term *F*_ts_ is the tip–sample force, which is a function of both the tip–sample distance, *d*, and velocity, 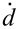. Here however, we focus on conservative forces since these are present even with gentle interactions. Hence we can write *F*_ts_(*d*). Since the higher harmonic amplitudes here are externally excited, the number of harmonics *N* that is to be monitored can, in principle, be arbitrarily chosen up to the limits of frequency detection, i.e., of the order of MHz, without compromising detection. The main constraint is that the number of higher modes, *M*, that is to be considered needs to be consistent with the number of higher harmonics *N* that are to be analysed [22]. For simplicity, we consider *M* = 2 and *N* = 10 in the numerical analysis without loss of generality. For clarity we emphasize that *M* is the number of modes and *N* is the number of harmonics taken into consideration in the analysis in this work. A particular mode or harmonic is referred to in lower case, i.e., *m* or *n* respectively.

The *n*th harmonic velocity 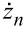 is

[3]
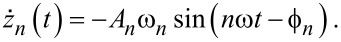


Multiplying Equation 1 by Equation 3 and integrating over a cycle results in

[4]
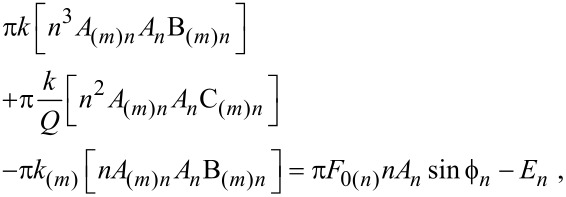


where 1 is assumed when no subscripts are given. The relationships (ω/ω_(_*_m_*_)_)^2^ = *k*/*k*_(_*_m_*_)_ and *Q*/*Q*_(_*_m_*_)_ = *ω*/*ω*_(_*_m_*_)_ [7] have been employed in Equation 4 and it has been assumed that the fundamental drive frequency ω is set near ω_(1)_. Furthermore, in Equation 4 *A*_(_*_m_*_)_*_n_* and *A_n_* are the amplitudes of the *n*th harmonic that correspond to the position of mode *m*, i.e., *z*_(_*_m_*_)_, and to the absolute position of the tip, i.e., *z*, respectively. Also

[5]
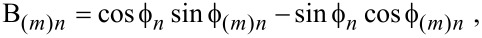


[6]
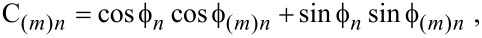


[7]
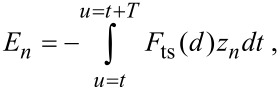


where 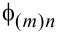 and 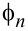 are the phase shifts of the *n*th harmonic that correspond to the *m*th mode position and the absolute position, *z*, respectively, and *E_n_* is the energy involved with the *n*_th_ harmonic tip–sample interaction. Near the modal frequency ω_(_*_m_*_)_ only the *m*th mode significantly contributes to the interaction and B_(_*_m_*_)_*_n_* ≈ 0 and C_(_*_m_*_)_*_n_* ≈ 1 in Equation 4. This approximation has been currently employed in the literature [6]. Nevertheless, far from the modes, these terms might not be zero. To allow for simple analytical formulae and ease the qualitative interpretation we consider the harmonics close to the modes only [6]. Then

[8]
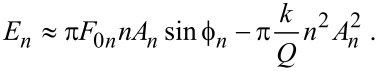


If the *n*th drive F_0_*_n_* is zero, then

[9]
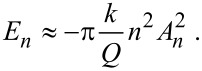


Equation 9 is the energy transferred to the *n*th harmonic of the cantilever through the tip–sample interaction. It should be noted that this is consistent with a conservative tip–sample force *F*_ts_(*d*) since the energy is provided during each cycle by the external driving force(s). The quadratic dependence of the energy *E_n_* on *nA_n_* is of particular relevance for the detection of higher harmonics. First, Equation 9 implies that for a given amplitude *A_n_* the transfer of energy *E_n_* scales quadratically with the harmonic number. This explains why for sufficiently large *n*, higher harmonics are typically undetectable. Second, the proportionality between *E_n_* and 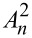 in Equation 9 explains why for higher harmonic amplitudes to be detected, the interaction in Equation 7 needs to be considerably large, even when *n* is not necessarily very large.

**Figure 1:**
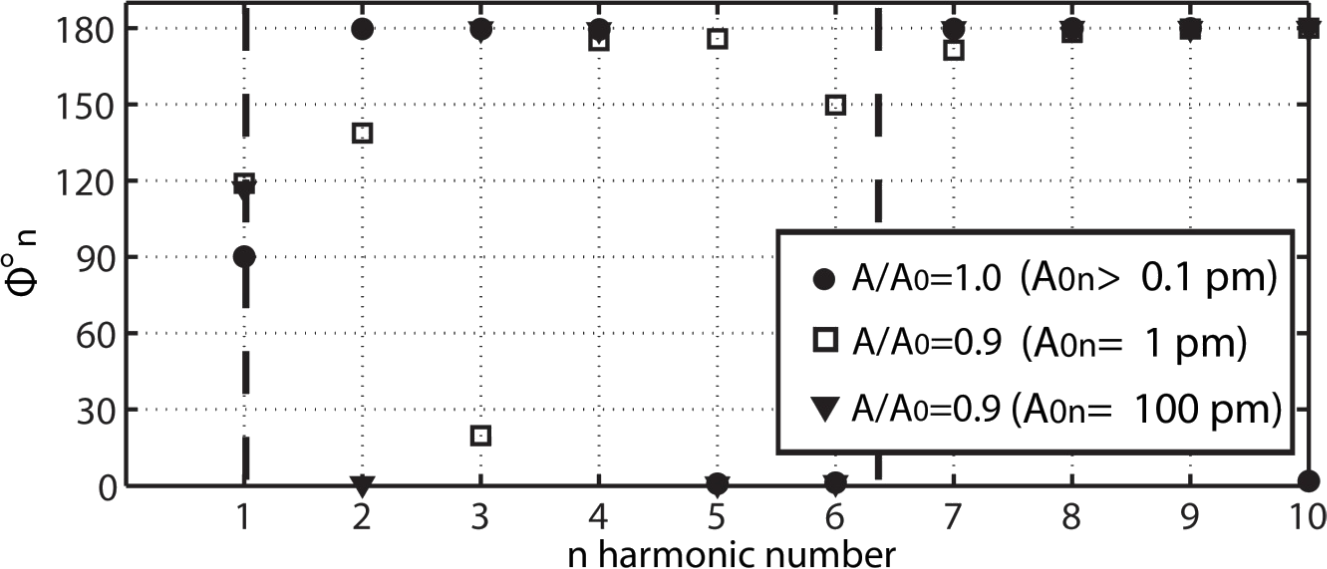
Phase shifts 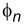 of higher harmonics, including the fundamental shift 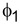, when *N* = 10 external harmonic drives are introduced. The values 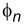 are shown for a free oscillating cantilever (circles). For the free cantilever the separation is *z*_c_ >> *A*/*A*_0_ = 1. Then the cantilever is gently interacting (peak forces smaller than 20 0pN) with the surface, i.e., *A*/*A*_0_ < 1, while the free higher harmonic amplitudes *A*_0_*_n_* are set to 1 (squares) and 100 (triangles) pm.

From Equation 8 it follows that *A_n_* can be set to any arbitrary value by increasing *F*_0_*_n_*, even if there is no tip–sample energy transfer, i.e., *E_n_* = 0. The higher harmonics for the free cantilever are termed *A*_0_*_n_*. This case corresponds to a free cantilever oscillating sufficiently high above the sample (*A*/*A*_0_ = 1) as illustrated in Figure 1 (circles). The data has been acquired by numerically solving the simultaneous equations in Equation 1 for the first two flexural modes, i.e., *M* = 2, and for *N* = 10. Furthermore, since only long range attractive forces are of interest here, the tip–sample force is simply [23]

[10]
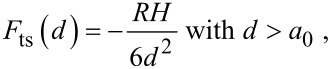


where *R* is the tip radius, *H* is the Hamaker constant and *a*_0_ is an intermolecular distance (*a*_0_ = 0.165 nm throughout and in all the data here, we consider *d* > *a*_0_ throughout). It is relevant to note that the Hamaker constant depends on the tip and sample in the sense that its value is determined by the atomic composition or chemical elements that compose the tip and the sample [40–41]. For this reason, in this work we will employ the terms chemistry, Hamaker and tip–sample composition or chemistry interchangeably. The common parameters in this work are *k* = 2 N/m, *Q* = 100, ω = 2π·70 kHz and *R* = 7 nm, i.e., they correspond to commercially available standard probes for AM AFM. Furthermore, in Figure 1, *H* = 6.2 × 10^−19^ J, i.e., it is close to that calculated for materials such as polystyrene or fused quartz [40]. The parameters for the second mode have been obtained with the above formulae [7]. The modal frequencies 1 and 2 are shown with dashed lines. The phase shifts 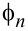 are shown in the vertical axis in Figure 1 for each harmonic. The actual harmonic amplitudes *A*_n_ that resulted when interacting are not shown, instead *A_n_* ≈ *A*_0_*_n_* is given throughout. The case of a free cantilever (circles) shows that the fundamental phase shift 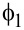 is exactly 90 degrees as expected while the higher harmonic phase shifts 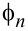 (*n* > 1) lie either close to 180° or to 0°. This is in agreement with Equation 8 when *E_n_* ≈ 0 since then

**Figure 2:**
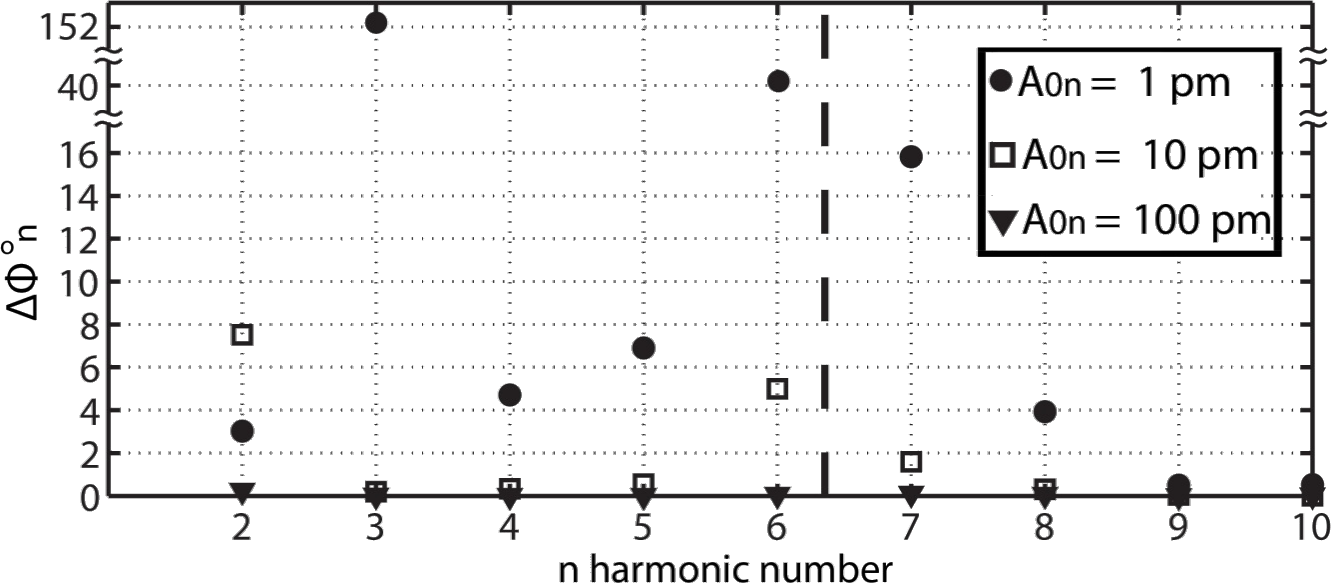
Phase shift analysis, in which the contrast in the higher harmonic phase shifts Δ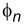 = abs(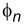
(*H*_2_) − 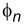
(*H*_1_)), *n* = 2–10, which is induced by variations in the Hamaker constant *H* is shown. The variation in *H* is *H*_2_ − *H*_1_ = 1.0 × 10^−19^ J, where *H*_2_ = 1.2 × 10^−19^ J, and effectively corresponds to variations in chemistry only. Results are shown when higher harmonic amplitudes *A*_0_*_n_* of 1 (circles), 10 (squares) and 100 (triangles) pm are introduced. Peak forces are smaller than 200 pN throughout.

**Figure 3:**
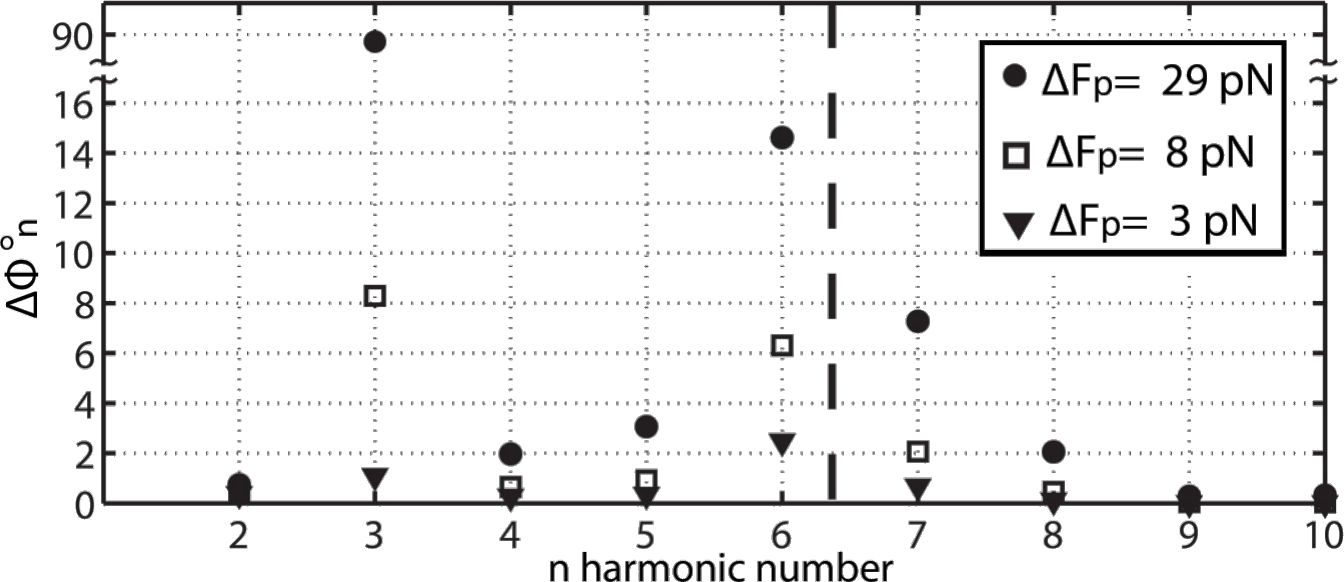
Phase shift analysis, in which the contrast in the higher harmonic phase shifts Δ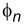 = abs(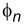
(*H*_2_) − 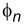
(*H*_1_)) for *n* = 2–10 results only from variations in the Hamaker constant, *H*, or in the chemistry. The variations of *H* are *H*_2_ − *H*_1_ = 0.2 × 10^−19^ J for *H*_2_ = 0.4 × 10^−19^ J (circles), 0.8×10^-19^ J (squares) and 1.2 × 10^−19^ J (triangles). These variations induce variations in peak force of 29 (circles), 8 (squares) and 3 (trinagles) pN.

[11]
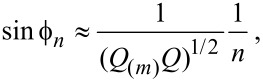


where the approximation *F*_0_*_n_* ≈ *k*_(m)_*n*^2^*A*_0_*_n_* (near *m*) has been employed. Also from Figure 1 (circles) it follows that for a free cantilever, and when *n* is higher than the modal frequency (close to a given mode and for *n* > 1), 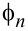 ≈ 180°. When *n* is lower than the modal frequency 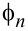 ≈ 0°. This is true irrespective of the value of *A*_0_*_n_*. When the tip is allowed to interact with the sample *E_n_* ≠ 0 and, from Equation 8, the phase shift 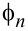 is affected by the interaction. Nevertheless, the weight of the driving force, i.e., the first term in Equation 8, increases with increasing *F*_0_*_n_*, or *A*_0_*_n_*, and then the sensitivity of 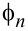 to *E_n_* might be compromised. This is confirmed in Figure 1 by allowing a gentle interaction, i.e., *A*_01_ ≡ *A*_0_ = 4 nm and *A*/*A*_0_ ≈ 0.9 (also Figure 2 and Figure 3), and monitoring 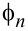
when A_0_*_n_* = 1 pm (squares) and A_0_*_n_* = 100 pm (triangles). When *A*_0_*_n_* = 100 pm (triangles) all 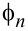 remain close to 180° or 0°. A shift in phase, i.e., from 180° to 0°, is observed for *n* = 2 only. While these jumps of nearly 180° might be of interest they are ignored from now on. The reader can refer to recent works that discuss multiple regimes of operation in bimodal AFM [31,33]. It follows that variations in Hamaker are not detected by higher harmonic frequencies when *A*_0_*_n_* = 100 pm. When *A*_0_*_n_* = 1 pm (squares), however, the values of 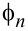 are not exactly 180° or 0° for some *n*. Thus, the values 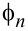 are now sensitive to the Hamaker values or tip–sample forces. The peak forces were 140 pN (circles) and 160 pN (triangles) respectively.

The loss of phase sensitivity to Hamaker variations with increasing *A*_0_*_n_* is further corroborated with the use of Figure 2 and by varying the Hamaker values from *H*_1_ = 0.2 × 10^−19^ J to *H*_2_ = 1.4 × 10^−19^ J, and setting *A*_0_*_n_* = 1 pm (circles), *A*_0_*_n_* =10 pm (squares) and *A*_0_*_n_* = 100 pm (triangles). This range of *H* is characteristic of materials interacting in ambient conditions [40]. The *y*-axis stands for the contrast in higher harmonic phase Δ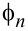 = abs(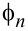
(*H*_2_) − 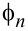
(*H*_1_)). We consider that variations, for which Δ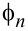 > 0.2° lie above the noise of the instrument and can potentially be detected. The corresponding variations in peak forces were 63, 47 and 79 pN respectively. The sensitivity of Δ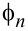 is clearly controlled by the chosen values of *A*_0_*_n_*_._ For example, if *A*_0_*_n_* = 100 pm then Δ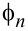 < 0.2° throughout. If *A*_0_*_n_* = 1 or 10 pm, however, then Δ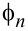 > 0.2° at least for some *n*. In particular, if *A*_0_*_n_* = 1 pm then Δ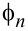 > 0.2° for all *n*. This implies that all the externally excited higher harmonics act as simultaneous contrast channels that are sensitive to Hamaker, or chemical, variations.

In Figure 3 the sensitivity of Δ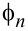 when *A*_0_*_n_* = 1 pm is tested by varying *H* (a) from *H*_1_ = 0.2 × 10^−19^ J to *H*_2_ = 0.4 × 10^−19^ J (peak force variation of 29 pN, circles), (b) from *H*_1_ = 0.6 × 10^−19^ J to *H*_2_ = 0.8 × 10^−19^ J (peak force variation of 8 pN, squares) and (c) from *H*_1_ = 1.2 × 10^−19^ J to *H*_2_ = 1.4 × 10^−19^ J (peak force variation of 3 pN, triangles). The shifts Δ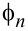 are larger than 0.2° for all *n* provided the variations in peak force are large enough (circles). If the variations in the peak force are sufficiently small then Δ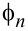 > 0.2° for some *n* only. Also, it can be deduced by inspection that, in general, Δ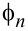 escalates with variations in peak force and changes non-linearly with variations in Hamaker since *H*_2_ − *H*_1_ = 0.2 × 10^−19^ J throughout in the figure. In fact, from Figure 3, the total contributions to the phase shift calculated as the sums ΣΔ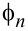 (*n* = 1–9) are 119.8, 19.3 and 5.4° and decrease with decreasing the variations in peak force, i.e., 29, 8 and 3 pN, respectively.

**Figure 4:**
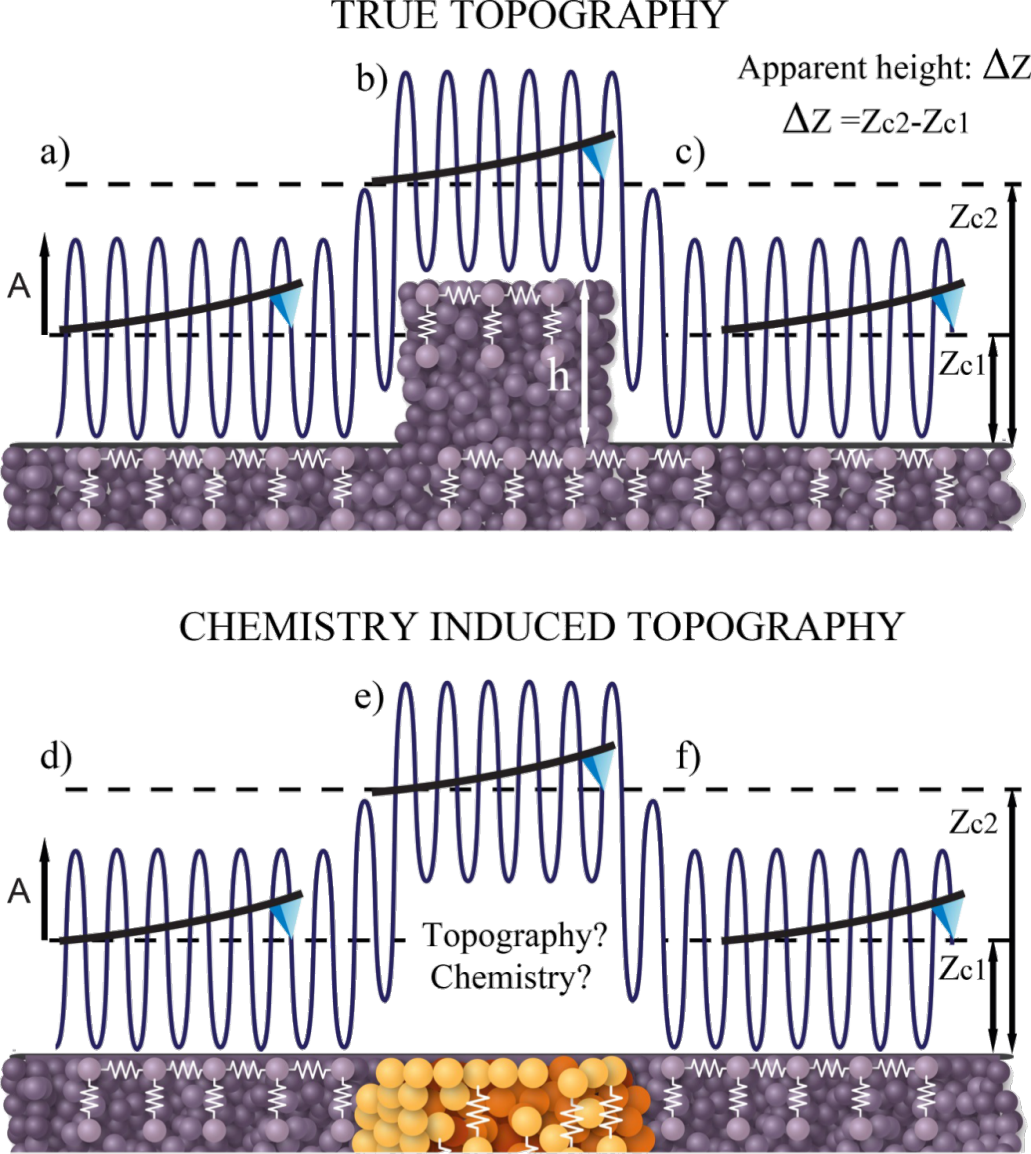
(a–c) Illustration of a cantilever oscillating above a surface and recovering the true height Δ*z*_c_ = *h* when there are no compositional heterogeneity or chemical variations. (d–f) Topographical variations Δ*z*_c_ > 0 nm induced by chemical or another compositional heterogeneity. The two cases can be decoupled by noting that it is only a compositional heterogeneity, if the phase shifts of higher harmonics, Δ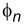, are non-zero.

It is also interesting to note that the source of variations in peak force with variations in Hamaker *H* (Equation 10), i.e., van der Waals forces, relates to variations in the distance of minimum approach, *d*_m_, with variations in *H*. To be more specific, *d*_m_, increases with increasing *H*. For example, in the simulations, by varying *H* from *H*_1_ = 0.2 × 10^−19^ J to *H*_2_ = 1.4 × 10^−19^ J the variation is Δ*d*_m_ ≈ 0.83 nm. This would experimentally result in a chemistry-induced apparent topography of approximately Δ*z*_c_ ≈ 0.83 nm. In standard AM AFM, in which a single frequency is externally excited, this apparent topography cannot be distinguished from true topography in the presence of conservative forces only (Figure 4). A true topography can only be reconstructed from AM AFM results, if there is a variation in topography only (Figure 4a). This means that the composition of the sample is homogeneous throughout. In particular, the above discussion indicates that variations in *H*, or chemistry alone, produce variations in apparent topography in AM AFM, for which Δ*z*_c_ > 0 nm (Figure 4b). The excitation of higher harmonics, however, provides experimental observables to differentiate between the two cases. Namely, the true reconstructed topography results only if Δ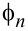 = 0° for all *n*. That is, if Δ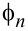 > 0°, even for a single *n*, there is a contribution to apparent topography induced by chemistry or other compositional variations.

### Thermal noise and higher harmonic external drives

As stated in the introduction, it has long been known that under ambient conditions higher harmonic amplitudes might be too small to be detected [3,15,42]. This is particularly true when monitoring higher harmonics and simultaneously applying gentle tip–sample forces [23]. In liquid environments, however, the second harmonic amplitude might be large enough [43] to be recorded to map the properties even of living cells [44–45]. Still, even in highly damped environments, harmonic amplitudes rapidly decrease with increasing harmonic number particularly when imaging soft matter [6,15,46]. The main discussion above has focused on externally driving higher harmonics to amplitudes that could be experimentally detected. Then, once these amplitudes are sufficiently high, the phase shifts Δ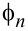 have been employed to map the composition through variations in the tip–sample Hamaker constant, *H*, in Equation 10. In this section, the presence of thermal noise is discussed with respect to the contrast in amplitude Δ*A_n_* and phase Δ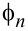 in the presence and absence of external drive forces at the higher harmonics frequencies.

First an example of the magnitude of the harmonic amplitudes and respective phase shifts that would result when higher harmonics are not externally excited is given (Table 1). In order to sense long-range forces only, the cantilever is driven with relatively small amplitudes, i.e., *A*_0_ = 4 nm and *A*/*A*_0_ = 0.9 as in the examples above. The harmonic amplitudes *A*_n_ are given in pm. Two examples for the amplitude response are shown, one for amplitudes resulting from *H*_1_ = 0.2 × 10^−19^ J (top row) and one for *H*_2_ = 1.4 × 10^−19^ J (second row). For *H*_1_, *A*_2_ is approx. 4 pm whereas *A*_3_ and *A*_6_ are approx. 1 pm. All other higher harmonics lie below 1 pm. For *H*_2_, *A*_2_ is approx. 3 pm and all other higher harmonics have values below 1 pm. The difference in amplitudes Δ*A_n_* = *A_n_*(*H*_2_) − *A_n_*(*H*_1_) that results from the variation in *H* is also given in the table. Only the second harmonic results in variations above 1 pm. Practically, these results imply that while higher harmonic amplitudes depend on the value of the Hamaker constant, or sample composition, the amplitude values are typically in the order of 1 pm or fractions of a pm. This is also true for variations in higher harmonic amplitudes Δ*A_n_*. The corresponding phase shifts 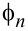 and variations in phase shifts Δ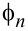 are also shown in Table 1 for *H*_1_ and *H*_2_. These are of the order of a hundredth of a degree or less except for sufficiently high harmonic numbers, i.e., *n* = 9 and 10. The amplitudes for these higher harmonics, however, are of the order of tens of femtometers or less.

**Table 1:** Harmonic amplitudes *A_n_* and the corresponding phase shifts 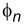 that result from Hamaker values of *H*_1_ = 0.2 × 10^−19^ J and *H*_2_ = 1.4 × 10^−19^ J. The differences in amplitudes Δ*A_n_* and phases Δ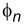 are also shown. A single external drive force has been employed (the fundamental frequency) and no thermal noise has been allowed.

	*A*_1_	*A*_2_	*A*_3_	*A*_4_	*A*_5_	*A*_6_	*A*_7_	*A*_8_	*A*_9_	*A*_10_

*A*_n_ [pm] for *H*_1_	3600.00	4.38	1.03	0.21	0.16	1.11	0.39	0.12	0.06	0.03
*A*_n_ [pm] for *H*_2_	3600.00	3.31	0.57	0.08	0.05	0.23	0.06	0.01	0.00	0.00

	Δ*A*_1_	Δ*A*_2_	Δ*A*_3_	Δ*A*_4_	Δ*A*_5_	Δ*A*_6_	Δ*A*_7_	Δ*A*_8_	Δ*A*_9_	Δ*A*_10_
Δ*A_n_* [pm]	0.00	−1.07	−0.46	−0.12	−0.12	−0.89	−0.33	−0.11	−0.05	−0.03

	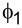	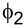	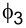	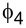	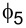	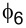	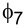	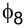	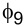	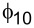
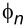 [°] for *H*_1_	115.83	141.25	167.20	12.77	39.91	66.18	90.49	116.55	142.44	168.29
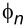 [°] for *H*_2_	115.84	141.25	167.20	12.77	39.90	66.18	90.48	116.52	142.29	167.78

	Δ 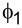	Δ 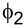	Δ 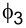	Δ 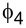	Δ 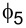	Δ 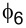	Δ 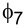	Δ 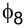	Δ 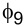	Δ 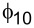
Δ 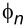 [°]	0.00	0.00	0.00	0.00	−0.01	0.00	0.00	−0.03	−0.14	−0.50

Thermal fluctuations are a fundamental source of intrinsic noise in atomic force microscopy [47]. Thus, while other sources of intrinsic and extrinsic noise should be acknowledged and might be present in a given experiment, thermal fluctuations are analyzed next in terms of their effects on amplitude and phase shifts. This should provide a measure of the impact of thermal noise on the enhanced contrast reported in this work (Figure 2 and Figure 3). Other technical issues such as tilt and probe geometry have also been ignored for simplicity since these typically involve a correction factor [48]. As in the work of Butt and Jaschke [47], the equipartition theorem is employed to estimate the thermal noise present in a given mode. However, since higher harmonics are discussed here, particular emphasis should be given to the noise at the frequencies of interest, i.e., at exact harmonic frequencies, and the noise in the detection bandwidths of interest. Then, the thermal noise power Δ*P*_TN_(Δ*f*) in the detection bandwidth of interest, Δ*f*, can be defined as

[12]
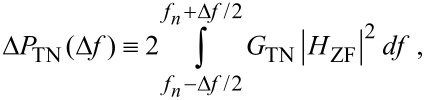


where TN stands for thermal noise, *f_n_* is the frequency of interest (*ω_n_* = 2π*f_n_*), that is the frequency of a particular harmonic *n*, *G*_TN_ is the power spectral density due to thermal noise, and |*H*_ZF_|^2^ is the modulus of the squared transfer function of a particular mode *m* of position *z_m_* relative to thermal force *F*_TN_. If *G*_TN_ is assumed to be constant for the bandwidth of interest in AFM experiments, i.e., f = 10^2^–10^6^, it follows from Equation 1 that the thermal energy in a given mode *m*, by invoking the equipartition theorem, is

[13]
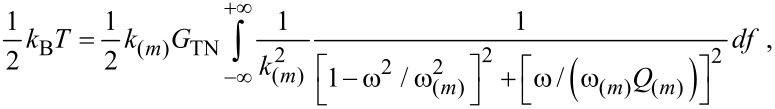


where here *T* = 300 K throughout, *f*_(_*_m_*_)_ is the natural resonant frequency of mode *m* in Hz and *df* = (*f*_(_*_m_*_)_/ω_(_*_m_*_)_)*dω*. Then

[14]
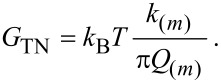


From Equation 13 and Equation 14, the thermal noise power in the detection bandwidth of interest, Δ*P*_TN_(Δ*f*), is found to be

[15]
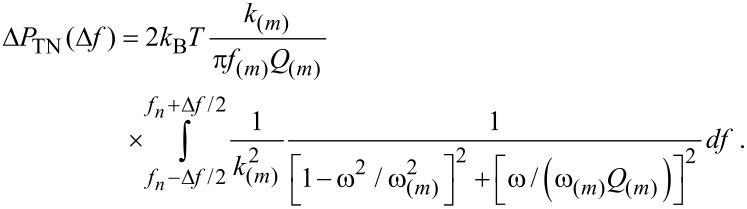


Finally, the associated amplitude due to thermal noise *A*_TN_ in the detection bandwidth Δ*f* is

[16]
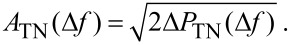


It should be noted that *A*_TN_ gives the contribution of thermal noise to the amplitude of a given mode *m* only. Each modal contribution of thermal noise to the amplitude should be calculated separately for each frequency in the formalism developed here. A driving force, *F*_TN_, can also be associated to thermal noise and the respective amplitude, *A*_TN_, (Equation 16) through a standard expression [49]

[17]
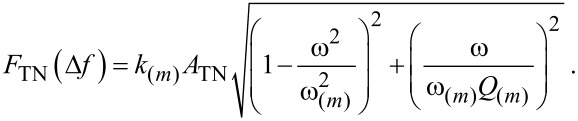


Equation 17 gives the effective drive force *F*_TN_ due to thermal fluctuations that should be expected for a given detection bandwidth Δ*f* and a given mode *m*. Since the upper boundaries for noise will be considered here, the phase of the thermal noise signal has been set to be in quadrature with respect to the external drive, i.e., either the fundamental external drive or the higher harmonic external drives when these are present. Focus is now placed on the harmonics *n* = 1, 2, 3 (close to the fundamental frequency of mode 1) and 6 (close to the fundamental frequency of mode 2), since these are sufficiently close to a given mode that only the contribution of thermal noise to the amplitude from a single mode needs to be considered. This simplifies the following discussion.

In Table 2 the amplitudes *A*_TN_*_n_* and forces *F*_TN_*_n_* calculated for three different values of detection bandwidth Δ*f* (5 kHz, 2 kHz and 0.2 kHz) are shown for *n* = 1, 2, 3 and 6. The values have been computed with the use of Equation 16 and Equation 17, with frequencies centered at the harmonic frequencies *f_n_*, for a given detection bandwidth Δ*f*. It is interesting to note that *A*_TN1_ lies between 44 and 19 pm for the three choices of detection bandwidth. These values are in agreement with those expected from an analysis that implies that all the thermal noise is centered exactly at resonance [47]. This is because the *Q* factors are relatively high (*Q*_1_ = 100 and *Q*_2_ = 600). The values of the thermal-noise amplitude expected at harmonics 2, 3 and 6 however are of the order of 0.1–1.0 pm.

**Table 2:** Amplitudes *A*_TN_*_n_* resulting from thermal noise for *n* = 1, 2, 3 and 6 and respective drive forces *F*_TN_*_n_* for detection bandwidths Δ*f* of 5, 2 and 0.2 kHz.

Δ*f* [kHz]	*A*_TN1_ [pm]	*F*_TN1_ [pN]	*A*_TN2_ [pm]	*F*_TN2_ [pN]	*A*_TN3_ [pm]	*F*_TN3_ [pN]	*A*_TN6_ [pm]	*F*_TN6_ [pN]

5	62.23	1.27	0.42	2.83	0.17	2.83	0.42	2.83
2	56.57	1.13	0.28	1.70	0.14	1.70	0.28	1.70
0.2	26.87	0.57	0.08	0.57	0.04	0.57	0.07	0.57

The effects that the thermal noise amplitudes in Table 2 have on the enhanced contrast reported in this work have been analyzed by adding the associated thermal noise forces, also shown in Table 2, to the equation of motion in Equation 1. The discussion below focuses on the values obtained for Δ*f* = 2 kHz in Table 2 since this is a detection bandwidth of practical relevance in standard AFM experiments [50].

The sensitivity of the phase shift to noise and signal can be defined here, and for the purpose of phase shifts in AM AFM, as follows. First assume that noise is allowed according to Table 2 (Δ*f* = 2 kHz) for a given value of the Hamaker constant, *H*. Here both *H*_1_ = 1.4 × 10^−19^ J and *H*_2_ = 1.4 × 10^−19^ J have been used in the simulations. According to this, thermal noise alone should lead to a difference in phase shift Δ
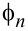
(*H*) = 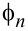
(*A*_TN_ > 0) − 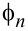
(*A*_TN_ = 0) for a given value of *H* since there is an effective driving force *F*_TNn_ due to thermal fluctuations (Table 2). The average of Δ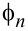 for the two Hamaker values can be taken as the noise in the phase signal as follows

[18]
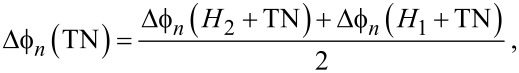


where TN stands for thermal noise as usual and Δ
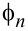
(TN) stands for the difference in phase shift at harmonic *n* that induced by thermal noise alone. Next the signal is defined as the phase shift induced by variations in Hamaker alone

[19]
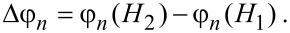


Finally, a parameter that quantifies the sensitivity of the phase shift to noise and signal, the phase ratio PR(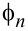) can be defined from the ratio between Equation 19 and Equation 18:

[20]
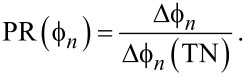


Large values of PR result in a high sensitivity of the phase shift to the signal, whereas low values of PR indicate a sensitivity of the phase shift to noise only. Three cases are discussed, which, for simplicity, focus on harmonics 2, 3 and 6 only and on *A*_0_*_n_* = 0, 1 and 10 pm.

**Case 1:** First, no higher harmonic external drives are allowed, which implies that *A*_0_*_n_* = 0 in Equation 2 for *n* > 1. This is the standard operational mode in dynamic AFM, in which a single external drive is employed. In this case we have PR = 0 throughout (Table 3).

**Case 2:** Higher harmonic external drives are allowed. In particular, *A*_0_*_n_* = 1 pm in Equation 2 for *n* > 1. This is the proposed mode of operation in this work. In this case we have PR > 1 throughout but the exact value depends on harmonic number (Table 3).

**Case 3:** Higher harmonic external drives are allowed. In particular, *A*_0_*_n_* = 10 pm in Equation 2 for *n* > 1. This is the proposed mode of operation in this work. When compared to case 2, however, the magnitudes of the external drives have been increased. In this case we also have PR > 1 throughout (Table 3).

**Table 3:** The phase ratio for a given harmonic phase shift *n*, PR(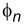), as defined by Equation 20 when 1) no higher harmonic external drives are allowed (*A*_0_*_n_* = 0) and when external drives lead to 2) *A*_0_*_n_* = 1 pm and 3) *A*_0_*_n_* = 10 pm.

	PR ( 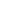 )	PR ( 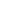 )	PR ( 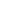 )

case 1: *A*_0_*_n_* =0	0.00	0.00	0.00
case 2: *A*_0_*_n_* =1 pm	1.90	22.09	7.29
case 3: *A*_0_*_n_* =10 pm	5.20	2.01	195.85

When looking at Table 3, one should recall that these are the upper-boundary values for noise since the phase of the thermal noise drives was set to be in quadrature. In summary, Table 3 shows that the phase ratio PR increases when external drives are applied at a given exact harmonic frequency, i.e., when *A*_0_*_n_* > 0. This is consistent with standard multifrequency operation, for which impressive results have already been obtained by exciting frequencies close to the resonant frequency of the second flexural mode [17,25–26]. In standard monomodal dynamic AFM, in which a single external drive is employed, the higher harmonics are excited by the tip–sample interaction according to Equation 9. That is, energy needs to flow into the higher harmonic frequencies in order to increase the amplitude signal. It is reasonable to assume that the increase in the sensitivity of the phase shift to the signal, i.e., the force, when external drives are applied is a consequence of energy both entering and leaving the given harmonic frequency of choice. That is, the fact that energy is supplied by the external drive at a given harmonic *n* implies that both positive and negative energy transfer might also occur at that frequency. Furthermore, when external drives are employed, this transfer occurs for a given phase shift that is now measured relative to the angle of the driving force. This is in agreement with the presence of the phase shift in Equation 8 and the absence of the phase shift in Equation 9 and might be related to the increase in the sensitivity of the phase shift to the tip–sample force as predicted here.

## Conclusion

In summary, we have introduced a method that makes readily accessible an arbitrary number of exact higher harmonics by externally driving them with amplitudes above the noise level. Driving with exact higher harmonics does not introduce sub-harmonic frequencies to the motion and the amplitudes do not significantly decay when the interaction is gentle. Once higher harmonic amplitudes are accessible, one can also detect variations in higher harmonic phase shifts. In this work, variations in sample composition, or chemistry, here modelled through the Hamaker constant, have been shown to lead to variations in higher harmonic phase shifts and amplitudes. In particular, variations in the Hamaker constant of the order of 10^20^ J can induce higher harmonic phase shifts in the order of 10°. This is provided the higher harmonic amplitudes are small enough, i.e., about 1–10 pm. These small variations in phase shift would suffice to distinguish between metals such as gold, silver or copper [40]. Higher harmonic phase shifts also provide the means to decouple the true topography from an apparent topography, which is induced by compositional variations. Furthermore this outcome should still be valid in standard bimodal imaging. Overall, the proposed approach, and variations, might ultimately fulfil the promise of rapid chemical identification with multiple contrast channels while simultaneously exerting only gentle forces on samples. Still it has to be acknowledged that, experimentally, it is expected that technical issues might arise from the multiple excitation of exact frequencies and from the set-up required to detect variations in higher harmonic phase. In particular, the set-up would require the generation of exact harmonic external drives to bring the harmonic amplitudes above the noise level while keeping them small enough to provide enough phase contrast. This last point is relevant since it has been shown that higher harmonic amplitudes should remain in the sub-100-pm range for the higher harmonic phase shifts to be significantly large, i.e., above 0.2°, in response to variations in the tip–sample force. On the other hand, an analysis of thermal fluctuation that exploits the equipartition theorem has also indicated that thermal noise should be of the order of 0.1–1.0 pm close to the higher harmonics modes. The implication is that the working amplitudes should lie in the range of 1 to 100 pm. The noise analysis has also shown that there is an increase in sensitivity of the phase shift to the tip–sample force when frequencies are externally excited. Nevertheless, ultimately, only experimental practice, implementation, ingenuity and further theoretical advances in the field are to establish what the limits of this approach are.
